# Cardioprotective effects of silver fir (*Abies alba*) extract in ischemic-reperfused isolated rat hearts

**DOI:** 10.3402/fnr.v60.29623

**Published:** 2016-10-17

**Authors:** Gorazd Drevenšek, Mojca Lunder, Eva Tavčar Benković, Borut Štrukelj, Samo Kreft

**Affiliations:** 1 Faculty of Medicine, University of Ljubljana, Ljubljana, Slovenia; 2 Faculty of Mathematics, Natural Sciences and Information Technologies, University of Primorska, Koper, Slovenia; 3 Faculty of Pharmacy, University of Ljubljana, Ljubljana, Slovenia

**Keywords:** Abies alba, silver fir trunk extract, cardioprotective, rat heart, p-coumaric acid, protocatechuic acid

## Abstract

**Background:**

Silver fir trunk extract (SFTE) is a complex mixture of antioxidative polyphenols (lignans and phenolic acids) from the trunks of silver fir trees (*Abies alba*, lignum). In our previous study, we have shown that SFTE exerts strong antioxidative and protective effects against atherogenic, diet-induced arterial wall damage.

**Objective:**

The aim of the present study was to test the potential protective effects of SFTE and its compounds, two phenolic acids (p-coumaric and protocatechuic acids) in ischemia–reperfusion injury of isolated rat hearts.

**Design:**

Isolated hearts of Wistar rats aged 4–8 weeks were exposed to perfusion, ischemia, and reperfusion periods. The experiments were performed using the following five groups: control, SFTE (10 µg/L), SFTE (100 µg/L), protocatechuic acid, and p-coumaric. Aortas were isolated to measure vascular responses in the presence of N^ω^-Nitro-L-arginine.

**Results:**

SFTE dose-dependently reduced ischemic-reperfusion heart damage, which was indicated as the decrease in the lactate dehydrogenase (LDH) release rate and arrhythmias duration by 80% and an increase in coronary flow rate during the reperfusion period. Two tested compounds (p-coumaric and protocatechuic acids) acted less cardioprotective, since they decreased the duration of arrhythmias only by 40 and 45%, respectively, and did not decrease LDH release rates during the reperfusion period. Only p-coumaric acid increased coronary flow rates, whereas protocatechuic acid did not.

**Conclusions:**

We conclude that the SFTE exerted the strongest cardioprotective effect, whereas its constituents (the p-coumaric and protocatechuic acids) were less effective in inducing cardioprotection.

Silver fir trunk extract (SFTE) is a complex mixture of antioxidative polyphenols from the trunks of silver fir trees (*Abies alba*, lignum). In our previous studies, we observed that SFTE exhibited strong antioxidative properties *in vitro* in 2,2-diphenyl-1-picrylhydrazyl (DPPH) test and *ex vivo* in primary human peripheral blood mononuclear cells (1). It also protected guinea pig arteries from impaired functional responses and morphological changes due to an atherogenic diet (2). Its primary constituents include lignans and phenolic acids, including p-coumaric and protocatechuic acids (1). Polyphenols, obtained from plants of broad use, are known to induce a favorable endothelial response in hypertension and beneficial effects in the management of other metabolic cardiovascular risks (3). Previous research regarding p-coumaric acid revealed that it exerts protective effects against myocardial infarct size in rats (4). P-coumaric acid modulates glucose and lipid metabolism, potentially improving and treating metabolic disorders (5). In addition, p-coumaric (6, 7) and protocatechuic acids (8) are effective against low-density lipoprotein (LDL) peroxidation *in vitro*. Protocatechuic acid exhibits anti-inflammatory activity in dendritic cells (9) and is a potential antiatherogenic (10), antihypertensive (11), and antiobesity (12) agent.

On the contrary, it becomes increasingly clear that chronic diseases are not caused, treated, or prevented via a single molecular target; therefore, consumption of complex molecular mixtures such as SFTE might provide greater health benefits than therapy with a single agent (13–15).

In this study, we evaluated the potential cardioprotective effects of SFTE and its constituents, p-coumaric and protocatechuic acids, in the ischemic-reperfusion-induced injury of isolated rat hearts.

## Materials and methods

### Animals and study design

#### Silver fir trunk extract

The extraction procedure and detailed extract characterization, including chromatographic profiling, are described in our previous publications (1, 2).

#### Animals

Male (range, 240–370 g) and female (range, 200–340 g) Wistar rats 4–8 weeks of age were obtained from the Institute of Pharmacology and Toxicology from the Faculty of Medicine, Ljubljana. All animals were bred under constant conditions, including a temperature of 21–24°C and a regular 12 h/12 h circadian cycle. The rats were fed unlimited amounts of water and rat feed in the form of Altromin 1,324 pellets (Altromin Spezialfutter GmbH & Co. KG, Lage, Germany). The exclusion criteria were as follows: animals with an inappropriate body weight or age, weak animals, a coronary flow of less than 7 mL/min or greater than 12 mL/min, the appearance of arrhythmias at the beginning of the experiment, and troublesome preparation.

#### Isolated heart preparation and protocol

The rats were anesthetized using 20% urethane at a dosage of 0.7 mL per 100 g of body weight (Sigma-Aldrich, St. Louis, MO, USA). Afterward, they were intraperitoneally treated with heparin (1,500 IU per animal per 100 g body weight; Krka, Novo mesto, Slovenia). A thoracotomy was performed, and a cannula filled with cold Krebs–Henseleit (K–H) solution with heparin (2,500 IU per 100 mL) was introduced into the aorta above the semilunar valve. The K–H solution contained the following (mM): NaCl, 118.4; KCl, 4.7; NaHCO_3_, 25 (Merck, Darmstadt, Germany); MgSO_4_×7 H_2_O, 1.2 (Riedel-de Haën, Seelze, Germany); NaH_2_PO_4_, 1.2; CaCl_2_, 1.5; and glucose, 11.1 (Kemika, Zagreb, Croatia). The solution was continuously gassed with 95% O_2_ and 5% CO_2_ (pH 7.4 at 38.5°C). During preparation, the heart was washed via the aorta with cold K–H solution to decrease its contractility. The hearts were removed, mounted on a Langendorff's apparatus, and perfused with an oxygenated K–H solution under a constant pressure. The valves were closed via retrograde flow (due to the pressure of the perfusion solution), and the coronary arteries were perfused. A pressure catheter (SPR-524, Millar, Houston, TX, USA) was introduced through the left atrium and the mitral valve into the left ventricle. An electrocardiogram was recorded from the surface of the heart with two silver (AgCl) electrodes (ITIS, Slovenia) and placed in the direction of the electrical axis of the heart. The signals were preamplified (Preamplifier Dewetron, Slovenia). The hearts were protected with a heated glass cover and parafilm to maintain constant temperature (38°C) and humidity.

The experiments were performed using the following five groups: the control group, the SFTE (10 µg/L) group, the SFTE (100 µg/L) group, the 1 µM protocatechuic acid (Sigma-Aldrich Chemical, Steinheim, Germany) group, and the 1 µM p-coumaric acid (Sigma-Aldrich Chemical, Steinheim, Germany) group. Each experiment lasted for 120 min. In the control group, the hearts were perfused with an oxygenated K–H solution during the first 30 min (perfusion period). Thereafter, global zero-flow ischemia with complete flow cessation of the K–H solution was performed for 40 min (ischemic period). The hearts were then perfused with the oxygenated K–H solution for 50 min (reperfusion period).

In the other four groups, the hearts were first perfused with an oxygenated K–H solution for 20 min followed by 10 min of perfusion with an oxygenated K–H solution including the test substances. Ischemia and reperfusion with the addition of test substances persisted for the same amount of time as the control group (16).

### Measured parameters

Coronary flow rate, lactate dehydrogenase (LDH) release rate, heart rate, arrhythmia incidence and duration, and left ventricular pressure protocols followed previously published procedures (17). The parameters were measured during both the perfusion and reperfusion periods.

### Isolated aorta preparation and protocols

The rats were sacrificed in a CO_2_ atmosphere via the injection of heparin (1,000, i.e., per animal per 100 g of body weight; Krka, Novo mesto, Slovenia). The thoracic aorta was isolated, rinsed, dissected, and cleansed of both fat and connective tissue. It was carefully cut transversally into eight cylindrical rings (2–3 mm in length) to preserve the endothelium. The aortic rings were immediately mounted in standard organ baths filled with K–H solution consisting of the following reagents (in mM): NaCl, 117.8; NaHCO_3_, 23.8; KCl, 4.7; CaCl_2_, 2.5 (Merck, Darmstadt, Germany); MgSO_4_×7 H_2_O, 1.2 (Riedel-de Haën, Seelze, Germany); KH_2_PO_4_, 1.2; and glucose, 11.0 (Kemika, Zagreb, Croatia).

Following mounting, equilibration of the rings at a resting tension of 20 mN was performed for 90 min, which entailed periodic adjustments of the tension to its resting value, as well as changing the K–H solution every 15 min. The rings were then contracted using 60 mM KCl for approximately 30 min or until stable contraction was obtained. Then, the rings were rinsed with K–H solution for 10 min. KCl contraction and rinsing were each repeated until a stable outcome was achieved. Phenylephrine and acetylcholine (both Sigma-Aldrich Chemical, Steinheim, Germany) were each dissolved in distilled water. For the relaxation measurements, the rings were precontracted with 0.1 mM phenylephrine until the contraction achieved a plateau; the rings were then relaxed with 0.1 mM acetylcholine. The tension was measured following each interval.

The rings were equilibrated with K–H solution for 10 min and then contracted again with 100 µL of phenylephrine. After reaching the plateau, we examined the relaxation of the pre-contracted rings by adding eight cumulative concentrations of SFTE (0.01–1,000 µg/L) at 2-min intervals. The tension was measured following each interval.

The control experiments followed the same procedure but used K–H solution instead of SFTE.

In the third portion of the experiment, relaxation with SFTE was measured in the rings that were incubated in K–H solution with the addition of a nitric oxide (NO) synthase inhibitor N^ω^-Nitro-L-arginine (L-NNA) for 10 min and then contracted with 100 µL phenylephrine. Cumulative concentrations of SFTE were added as described above.

The vascular responses were processed and recorded using a Dewetron acquisition system (Dewetron, Graz, Austria) followed by analogue to digital conversion (NI PCI-6013; National Instruments, Austin, TX, USA) using DeweSoft 6.1 software (Dewetron, Trbovlje, Slovenia).

### Statistical analysis

Statistical analyses were performed using GraphPad Prism 6.0 (GraphPad Software, San Diego, CA, USA). The values are expressed as the means ±SEMs. The relative relaxation responses of the arterial rings are expressed as the percentages of the phenylephrine precontracted aortic rings. A two-way analysis of variance (ANOVA) test with Bonferroni's post-test was performed for intergroup comparisons. A *p*-value <0.05 was considered statistically significant.

## Results and discussion

In the present study, we found that SFTE possessed a strong cardioprotective effect in ischemia–reperfusion injury of isolated rat hearts, whereas its compounds, two phenolic acids (p-coumaric and protocatechuic acids), were less effective in inducing cardioprotection. In order to determine the degree of cardiac tissue injury, LDH release rates were analyzed in the coronary effluents. Coronary flow values, heart rates, left ventricular pressure trends, and arrhythmia occurrences were also measured in order to assess alterations in heart function.

**Fig. 1:**
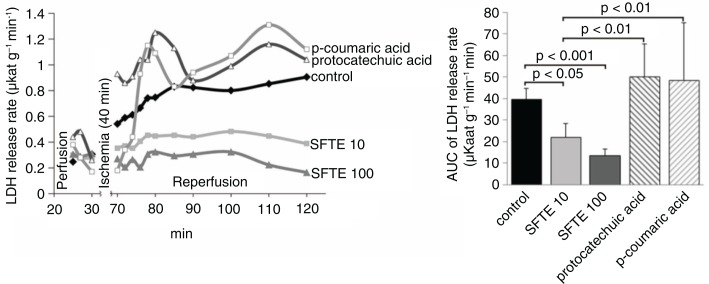
Lactate dehydrogenase (LDH) release rate from isolated rat hearts during both perfusion and reperfusion (left). Areas under the curve from the left diagram (reperfusion period) with standard errors (right). SFTE 10 and SFTE 100 represent silver fir trunk extracts at concentrations of 10 and 100 µg/L, respectively.

The evaluation of the LDH release rates revealed the severity of the ischemia and reperfusion-induced myocardial injuries. LDH is a cytoplasmic enzyme and is released in the setting of tissue damage, making it a relevant biomarker of both cell injury and disease. In the control group, the LDH release rates gradually increased in the course of the reperfusion period until the end of the experiments (Fig. 1). In the SFTE groups, the LDH release rates were significantly decreased compared to the control group (*p*<0.01 SFTE 10 and SFTE 100) during the reperfusion period and remained stable to the end of the experiments. In addition, LDH release rates were maximally decreased in the 100 µg/L SFTE group. One of the possible explanations for this finding could be the antioxidant activity of the constituent compounds and their cardioprotective and vasoactive effects, which were also noted in several studies involving pycnogenol (18, 19). P-Coumaric and protocatechuic acids did not exert these effects; therefore, other substances derived from SFTE are potentially responsible for the observed phenomenon.

**Fig. 2:**
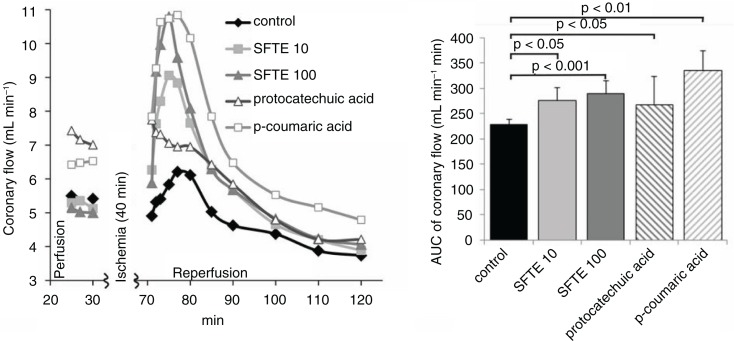
Coronary flow in the isolated rat hearts during both perfusion and reperfusion (left). Areas under the curve of the left diagram (reperfusion period) with standard errors (right). SFTE 10 and SFTE 100 represent silver fir trunk extracts at concentrations of 10 and 100 µg/L, respectively.

In the coronary flow analysis, we found that it increased significantly at the beginning of the reperfusion period in each of the groups, except in the protocatechuic acid group (Fig. 2). Higher concentrations of SFTE caused larger increases in coronary flow (*p*<0.05 SFTE 10, *p*<0.001 SFTE 100), and these increases were comparable with the effect of the p-coumaric acid. This finding is illustrative of the acute vasodilatory effects of SFTE and p-coumaric acid, but not protocatechuic acid in isolated hearts exposed to ischemic-reperfusion injury. It is interesting that the extract did not increase the coronary flow rate in the perfusion period (before ischemia), but only in the reperfusion period, which indicates protective effect in the ischemia–reperfusion injury, but not in basal conditions. The mechanism behind the vasodilatory effect of the extract is probably combined, partly due to the antioxidant activity and partly due to the effect on the NO synthase pathway. The latter is consistent with the results on isolated thoracic aortas, where we have shown that endothelial nitric oxide synthase (eNOS) inhibition completely abolished the vasodilatory effects of the extract. This observation suggests that the tested substances exhibit antioxidative properties in addition to vasodilatory properties.

**Fig. 3:**
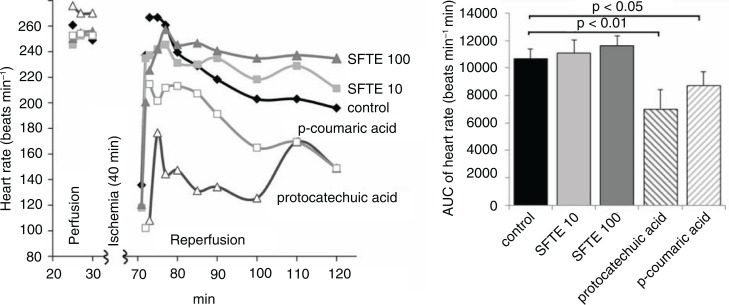
The rates of isolated rat hearts during both perfusion and reperfusion (left). Area under the curve of the left diagram (reperfusion period) with standard errors (right). SFTE 10 and SFTE 100 represent silver fir trunk extracts at concentrations of 10 and 100 µg/L, respectively.

**Fig. 4:**
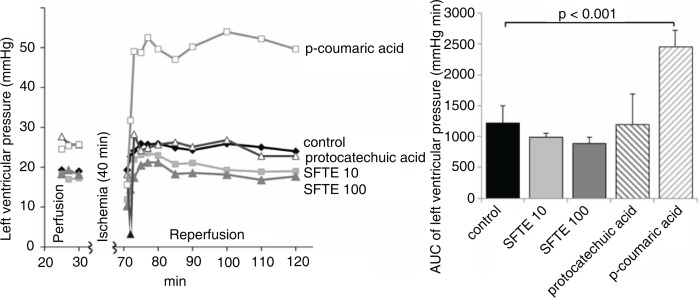
The left ventricular pressures of isolated rat hearts during both perfusion and reperfusion (left). Areas under the curve of the left diagram (reperfusion period) with standard errors (right). SFTE 10 and SFTE 100 are silver fir trunk extracts at concentrations of 10 and 100 µg/L, respectively.

The hearts treated with protocatechuic and p-coumaric acids exhibited lower rates compared with the control group (Fig. 3). The SFTE extract did not significantly influence the heart rate but appeared to restore the pre-ischemic heart rate values compared with the control group. In the latter, the heart rate gradually decreased in the reperfusion period. We observed no significant changes of left ventricular pressure values between the study groups (Fig. 4).

**Fig. 5:**
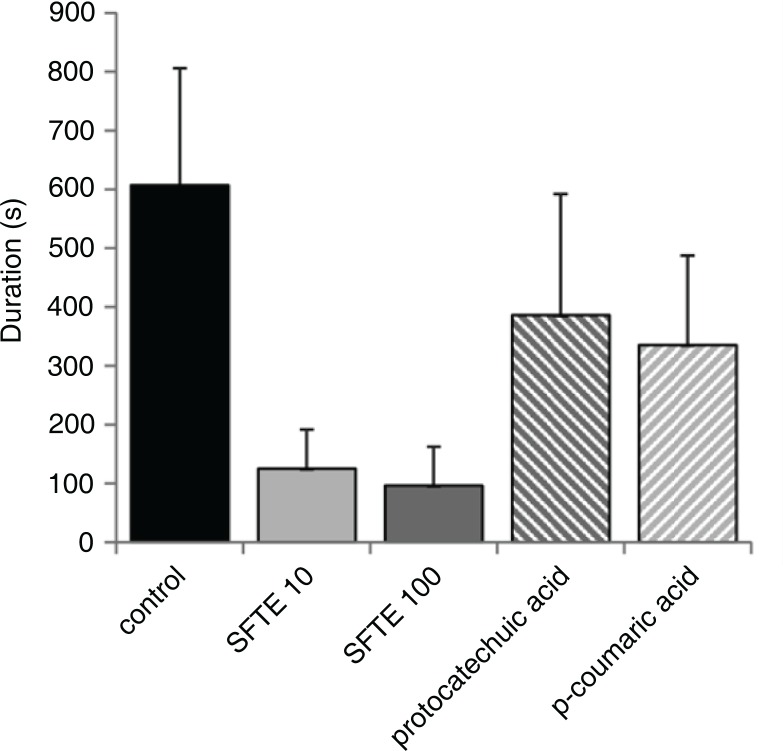
The total arrhythmia duration in isolated rat hearts during reperfusion, which followed ischemia. The hearts were either untreated (control) or perfused with 10 µg/L or 100 µg/L SFTE solution, 1 µM protocatechuic acid, or 1 µM p-coumaric acid.

**Fig. 6:**
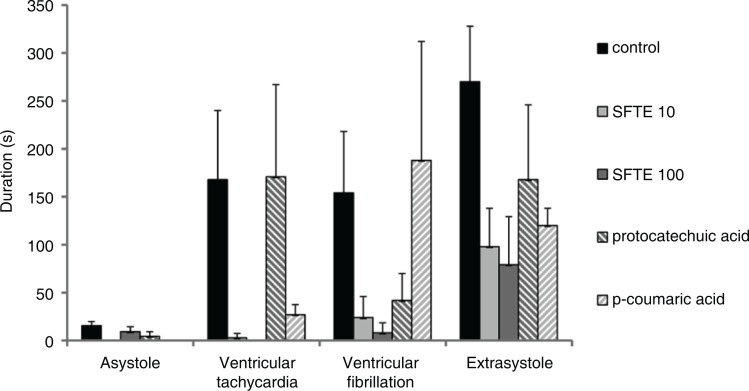
Durations of the individual types of arrhythmias in isolated rat hearts during reperfusion, which followed ischemia. The hearts were either untreated (control) or perfused with a 10 µg/L or a 100 µg/L SFTE solution, 1 µM protocatechuic acid, or 1 µM p-coumaric acid.

We also analyzed the duration of the arrhythmias in the reperfusion period, since we observed no arrhythmias in the perfusion period. The duration of arrhythmias was the highest in the control group of hearts not treated with phenols (Fig. 5). In the experiments in which the hearts were treated by either 10 µg/L or 100 µg/L of SFTE, the total duration of the arrhythmias was decreased by 80% (*p*<0.001) and persisted less than 120 sec. The reduction of arrhythmias duration achieved by p-coumaric and protocatechuic acids was smaller: only by 40% and 45%, respectively. We speculate that another substance derived from SFTE, more probably even the synergy of its constituents is responsible for the antiarrhythmic effect. A distinct decrease in the individual types of arrhythmias was noted in the SFTE groups (Fig. 6). Ventricular tachycardia durations were decreased at both concentrations (*p*<0.01). The decreases in ventricular fibrillation were dose dependent (*p*<0.05 SFTE 10, *p*<0.01 SFTE 100). The influence on extrasystole duration was the most significant effect observed (*p*<0.01 SFTE 10, *p*<0.001 SFTE 100). Nevertheless, the reductions in arrhythmia duration confirmed the cardioprotective effects of SFTE, which is less expressed in its phenolic acid constituents.

It is important to note that all the parameters in isolated heart experiments were measured only in the perfusion and reperfusion periods and not in the ischemia period. After the perfusion period, the hearts were exposed to the zero-flow ischemia in order to induce the ischemic damage to the heart. Ischemic phase lasted for 40 min, and during this phase of the experiment there was a complete flow cessation of the K–H solution to the heart. Therefore, during this phase observed parameters could not be measured, since there was no effluent through the heart. This methodology was previously described in more detail (17).

**Fig. 7:**
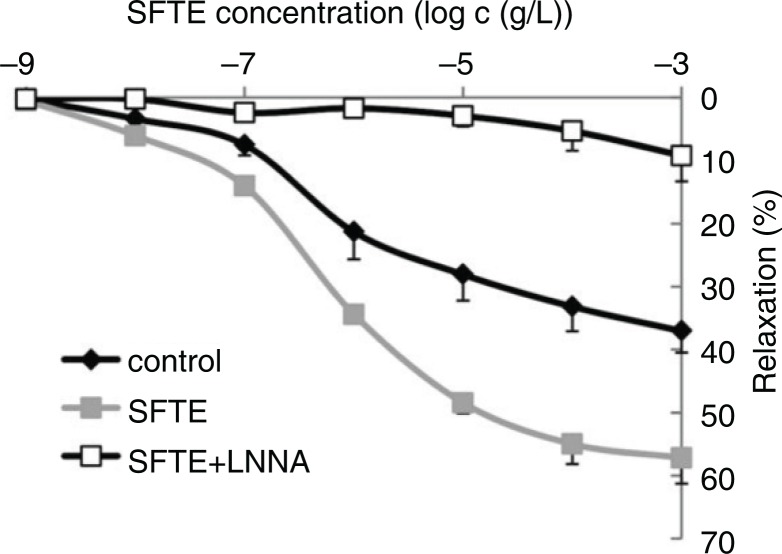
Aortic relaxation achieved with acetylcholine following precontraction with phenylephrine. SFTE enhanced the relaxation ability of the aortas; the effect was completely inhibited by the NO synthase inhibitor (L-NNA).

The relaxation ability of the isolated thoracic aortas with preserved endothelium was significantly increased in a concentration-dependent manner by the administration of SFTE (Fig. 7). The eNOS inhibitor L-NNA completely suppressed the relaxation ability of the aorta. The experiment revealed that the mechanism of relaxation by SFTE is mediated primarily via direct or indirect promotion of eNOS activity and the signaling pathway that accelerates the activity of the enzyme. These results are supported by the results of similar studies involving polyphenols (20–22). Pycnogenol studies also revealed that the relaxation is dependent on the endothelium (23).

In summary, we confirm the positive effects exerted by SFTE on ischemic-reperfusion injury in isolated rat hearts as well as the vascular protective effects of SFTE. Since the total extract had much higher effect than its constituents, p-coumaric and protocatechuic acids, we assume that they are involved but are not mainly responsible for its beneficial effects. Therefore, only SFTE can be used for the prevention of heart damage, presumably by the prevention of endothelial dysfunction, an important contributor to the development of atherosclerosis and other types of cardiovascular disease, which should be tested in future studies.

## References

[1] Benković ET, Grohar T, Žigon D, Švajger U, Janeš D, Kreft S (2014). Chemical composition of the silver fir (*Abies alba*) bark extract Abigenol^®^ and its antioxidant activity. Ind Crops Prod.

[2] Drevenšek G, Lunder M, Benković ET, Mikelj A, Štrukelj B, Kreft S (2015). Silver fir (*Abies alba*) trunk extract protects guinea pig arteries from impaired functional responses and morphology due to an atherogenic diet. Phytomedicine.

[3] Fernández-Arroyo S, Camps J, Menendez JA, Joven J (2015). Managing hypertension by polyphenols. Planta Med.

[4] Stanely MPP, Roy AJ (2013). p-Coumaric acid attenuates apoptosis in isoproterenol-induced myocardial infarcted rats by inhibiting oxidative stress. Int J Cardiol.

[5] Yoon S-A, Kang S-I, Shin H-S, Kang S-W, Kim J-H, Ko H-C (2013). p-Coumaric acid modulates glucose and lipid metabolism via AMP-activated protein kinase in L6 skeletal muscle cells. Biochem Biophys Res Commun.

[6] Kiliç I, Yeşiloğlu Y (2013). Spectroscopic studies on the antioxidant activity of p-coumaric acid. Spectrochim Acta A Mol Biomol Spectrosc.

[7] Cheng J-C, Dai F, Zhou B, Yang L, Liu Z-L (2007). Antioxidant activity of hydroxycinnamic acid derivatives in human low density lipoprotein: mechanism and structure–activity relationship. Food Chem.

[8] Yin M, Chao C (2008). Anti-Campylobacter, anti-aerobic, and anti-oxidative effects of roselle calyx extract and protocatechuic acid in ground beef. Int J Food Microbiol.

[9] Del Cornò M, Varano B, Scazzocchio B, Filesi C, Masella R, Gessani S (2014). Protocatechuic acid inhibits human dendritic cell functional activation: role of PPARγ up-modulation. Immunobiology.

[10] Wang D, Zou T, Yang Y, Yan X, Ling W (2011). Cyanidin-3-O-β-glucoside with the aid of its metabolite protocatechuic acid, reduces monocyte infiltration in apolipoprotein E-deficient mice. Biochem Pharmacol.

[11] Deng J-S, Lee S-D, Kuo W-W, Fan M-J, Lin Y-M, Hu W-S (2014). Anti-apoptotic and pro-survival effect of protocatechuic acid on hypertensive hearts. Chem Biol Interact.

[12] Kang S-W, Kang S-I, Shin H-S, Yoon S-A, Kim J-H, Ko H-C (2013). Sasa quelpaertensis Nakai extract and its constituent p-coumaric acid inhibit adipogenesis in 3T3-L1 cells through activation of the AMPK pathway. Food Chem Toxicol.

[13] Weseler AR, Bast A (2012). Pleiotropic-acting nutrients require integrative investigational approaches: the example of flavonoids. J Agric Food Chem.

[14] Guimarães R, Barros L, Carvalho AM, Ferreira ICFR (2011). Infusions and decoctions of mixed herbs used in folk medicine: synergism in antioxidant potential. Phytother Res.

[15] Caballero-George C (2015). Cardiovascular protection by natural products. Planta Med.

[16] Kuzner J, Drevensek G, Gersak B, Budihna M (2004). Hypoxic and pharmacological preconditioning preserves vasomotor response of porcine coronary artery. Pol J Pharmacol.

[17] Kuhar P, Lunder M, Drevensek G (2007). The role of gender and sex hormones in ischemic-reperfusion injury in isolated rat hearts. Eur J Pharmacol.

[18] Liu F, Lau BH, Peng Q, Shah V (2000). Pycnogenol protects vascular endothelial cells from beta-amyloid-induced injury. Biol Pharm Bull.

[19] Ozer ŞA, Şener G, Ercan F (2009). Protective effects of Pycnogenol against ischemia reperfusion-induced oxidative renal injury in rats. Ren Fail.

[20] Maimoona A, Naeem I, Saddiqe Z, Jameel K (2011). A review on biological, nutraceutical and clinical aspects of French maritime pine bark extract. J Ethnopharmacol.

[21] de Pascual-Teresa S, Moreno DA, García-Viguera C (2010). Flavanols and anthocyanins in cardiovascular health: a review of current evidence. Int J Mol Sci.

[22] Ziberna L, Lunder M, Moze S, Vanzo A, Tramer F, Passamonti S (2010). Acute cardioprotective and cardiotoxic effects of bilberry anthocyanins in ischemia-reperfusion injury: beyond concentration-dependent antioxidant activity. Cardiovasc Toxicol.

[23] Fitzpatrick DF, Bing B, Rohdewald P (1998). Endothelium-dependent vascular effects of Pycnogenol. J Cardiovasc Pharmacol.

